# The *Enhancer of split *and *Achaete-Scute *complexes of Drosophilids derived from simple ur-complexes preserved in mosquito and honeybee

**DOI:** 10.1186/1471-2148-5-67

**Published:** 2005-11-17

**Authors:** Rebekka Schlatter, Dieter Maier

**Affiliations:** 1 Universität Hohenheim, Institut für Genetik, Garbenstr. 30, 70599 Stuttgart, GERMANY

## Abstract

**Background:**

In *Drosophila melanogaster *the *Enhancer of split-Complex [E(spl)-C] *consists of seven highly related genes encoding basic helix-loop-helix (bHLH) repressors and intermingled, four genes that belong to the Bearded (Brd) family. Both gene classes are targets of the Notch signalling pathway. The *Achaete-Scute-Complex [AS-C] *comprises four genes encoding bHLH activators. The question arose how these complexes evolved with regard to gene number in the evolution of insects concentrating on Diptera and the Hymenoptera *Apis mellifera*.

**Results:**

In Drosophilids both gene complexes are highly conserved, spanning roughly 40 million years of evolution. However, in species more diverged like *Anopheles *or *Apis *we find dramatic differences. Here, the *E(spl)-C *consists of one bHLH (*mβ*) and one Brd family member (*mα*) in a head to head arrangement. Interestingly in *Apis *but not in *Anopheles*, there are two more *E(spl) *bHLH like genes within 250 kb, which may reflect duplication events in the honeybee that occurred independently of that in Diptera. The *AS-C *may have arisen from a single *sc/l'sc *like gene which is well conserved in *Apis *and *Anopheles *and a second *ase *like gene that is highly diverged, however, located within 50 kb.

**Conclusion:**

*E(spl)-C *and *AS-C *presumably evolved by gene duplication to the nowadays complex composition in Drosophilids in order to govern the accurate expression patterns typical for these highly evolved insects. The ancestral ur-complexes, however, consisted most likely of just two genes: *E(spl)-C *contains one bHLH member of *mβ *type and one Brd family member of m*α *type and *AS-C *contains one *sc/l'sc *and a highly diverged *ase *like gene.

## Background

The Notch pathway is one of the best studied cell to cell communication systems in the animal kingdom. It is highly conserved and used from worm to man. This pathway is needed whenever cell decisions are influenced by cell-cell communication, and also during proliferation or pathway crosstalk [1]. Using *Drosophila melanogaster *as a model system, the Notch pathway was intensely studied over many years. The best defined process governed by Notch is called "lateral inhibition": cells of a given fate are singled out from an equivalence group of the same fate, whereas the differentiation of the other cells is suppressed by the Notch signal. This happens for example during neurogenesis, where neuroblasts are selected from proneural clusters; they keep neural fate, whereas the surrounding cells eventually differentiate as epidermoblasts. The name giving transmembrane Notch-receptor interacts physically with the extracellular domain of the transmembrane ligands Delta or Serrate of the signalling cell. After this activation, the intracellular domain of Notch is cleaved and travels into the nucleus where it transcriptionally activates together with Suppressor of Hairless [Su(H)] genes of the *Enhancer of split *complex [*E(spl)-C*]. *E(spl) *gene products in turn repress the activity of proneural genes encoded for example by the *Achaete-Scute-Complex *[*AS-C*]. As consequence these cells stay undifferentiated to become epidermoblasts later on, whereas the signalling cell enters into the programmed neural cell fate. In the focus of our studies are these two complexes, *E(spl)-C *and *AS-C*, since in *D. melanogaster *they are composed of several genes with complex expression patterns and specific yet partly redundant functions.

**Figure 1:**
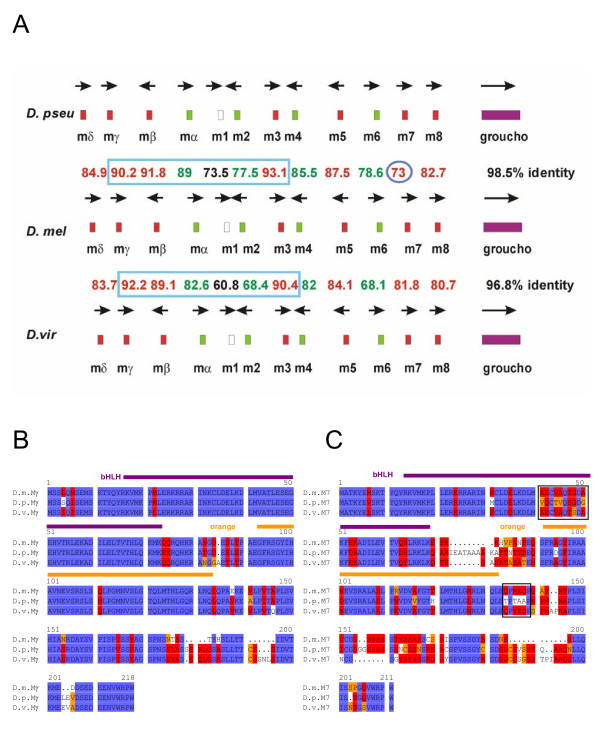
**Conservation of the *E(spl)-C *in Drosophilids**. A) The *E(spl)-C *is highly conserved in Drosophilids with regard to gene number and transcript orientation (arrows). The size of the complex is also almost the same; in *D. pseudoobscura *(*D. pseu*) it is only slightly larger than in *D. melanogaster *(*D. mel*). The smallest seems to be the *D. virilis *(*D. vir*) complex, however, the *virilis *sequence was not completed at the time. The best identity at protein level is found between the Gro orthologs (purple) followed by the bHLH proteins (red). Interestingly the proteins of the centrally located bHLH genes Mγ, Mβ and M3 (framed blue) are best conserved. Higher identities are seen between the *melanogaster *and *pseudoobscura *orthologs than between the ones of *D. melanogaster *and *D. virilis*, with the exception of M7 (blue circle). The worst conserved member of the complex is M1. (Numbers give % identity between the proteins). B) Alignment of the Mγ and (C) M7 orthologs. The bHLH (purple) and orange domains are the best conserved parts of the orthologs. The M7 sequences labelled with the black box are unexpectedly better conserved in *D. virilis *than in *D. pseudoobscura *compared with *D. melanogaster*. Identical residues are marked in blue; red shows highly related and yellow similar residues.

*Enhancer of split *was originally identified by genetic means as enhancer of the duplicated bristle phenotype found in the recessive *Notch *allele *split *[2]. In order to identify the responsible gene the *Enhancer of split *gene region has been cloned. In this region 13 transcription units are located and named *m1 *to *m10 *and *mα *to *mδ*. It was a surprise that seven of these genes encode structurally related proteins characterized by a basic and a helix-loop-helix domain (bHLH), a further alpha-helix forming 'orange domain' and a stereotypic terminus with the amino acids tryptophane, arginine, proline, tryptophane (WRPW). Later it was shown that this motif serves as binding site for the global co-repressor Groucho (Gro, transcription unit m9/10), which is encoded by a gene localised next to the bHLH gene cluster [3-10]. The bHLH genes *m3*, *m5*, *m7*, *m8*, *mβ*, *mγ *and *mδ *(see Fig. 1; [8]) are all transcriptional targets of Notch: they encode the effector proteins of the Notch signal at least in the process of lateral inhibition [1,11-13]. Apart from the seven bHLH genes and the neighbouring *gro *locus, the *E(spl)-C *comprises five further genes. Four genes *mα*, *m2*, *m4 *and *m6 *share structural similarity with the *Bearded (Brd)*-gene family and are themselves transcriptional targets of Notch, whereas *m1 *is completely unrelated and encodes a putative protease inhibitor [14,15]. Larger deficiencies encompassing several of *E(spl) *transcripts cause a severe neural hyperplasia, whereas loss of activity of single genes do not, suggesting redundancy of these seven bHLH genes [7,10,16-18]. However, remarkable differences were observed between the respective expression patterns in the embryo as well as in postembryonic tissues, arguing against complete redundancy [8,12,14,15,19-22]. Consistently, a high conservation of the entire complex was observed in the rather distantly related fly species *Drosophila hydei *[23]. The question, however, remains whether gene number and structure of the complex is conserved during longer terms of evolution. For example, in vertebrates an *E(spl)-C *like in *D. melanogaster *does not exist. Here the Notch target genes have been classified as HES (hairy/Enhancer of split) and HER/HESR (hairy/Enhancer of split related) genes, because the *D. melanogaster *segmentation gene *hairy *encodes a bHLH protein with orange domain and WRPW motif that is as similar to the vertebrate HES genes as are the *E(spl) *bHLH genes [24,25]. The vertebrate genes are not clustered in a complex. Apparently, in the course of evolution rearrangements occurred between these Notch target genes.

The *AS-C *in *D. melanogaster *comprises four genes, *achaete (ac)*, *scute (sc)*, *lethal of scute (l'sc) *and *asense (ase) *that all encode transcriptional activators of the bHLH class. They determine proneural fate and are thus required for the development of the central and peripheral nervous system [13,26-29]. These genes have been also studied in vertebrates. In the mouse there are three *achaete-scute *family members abbreviated ASH for Ac-Sc-Homolog [30]: MASH-1 and XASH-3/CASH-4 are two members that are involved in the development of the nervous system [30]. Both complexes are therefore good candidates to look for the changes that occurred during insect evolution.

In this work, we studied the evolution of the *E(spl)-C *and *AS-C *by making use of the recent advances in the genome projects of the Diptera *D. pseudoobscura*, *D. virilis*, *Anopheles gambiae *and the Hymenoptera *Apis mellifera*. The estimated distances are nearly 30 million years (Myr) separating *D. melanogaster *and *D. pseudoobscura *which both belong to the *Sophophora *subgenus and around 40 Myr separating the *Sophophora *from the *Drosophila *subgenus where *D. virilis *belongs to [31]. The distance between these modern dipterans and the more ancient ones like the Culicidae *A. gambiae *is estimated 200–250 Myr and that between Diptera and Hymenoptera like the honeybee *A. mellifera *250–300 Myr [32]. Our study shows that both gene complexes are highly conserved in Drosophilids with regard to overall size, gene number and structural similarity of the encoded proteins. In contrast, more ancient dipterans like the mosquito and similarly also the honeybee have much simpler gene complex structures: the *E(spl)-C *consists of just one *mβ *like bHLH gene and one *mα *like Brd-type gene. However, in *Apis *two more bHLH/WRPW coding genes are found within about 200 kb and may reflect an enlargement of the *E(spl)-C *in this species. The *AS-C *consists of only one *sc/l'sc *like member and one further *ase*-like gene that is, however, widely diverged. These data suggest that the evolution of modern Drosophilids included an enlargement of these complexes, notably a multiplication of the genes in the *E(spl)-C *that seem to have subsequently specified their roles in Notch signalling pathway. Their strict conservation in Drosophilids argues for a diversification presumably driven by their highly specified expression patterns and regulatory activities.

## Results

### The *Enhancer of split *complex in *Drosophilids*

The *Enhancer of split *complex [*E(spl)-C*] consists of 13 transcription units (Fig. 1): seven genes (*m3*, *m5*, *m7*, *m8*, *mβ*, *mγ*, *mδ*) encode highly related basic helix-loop-helix proteins, four have been grouped to the Brd-family (*m2*, *m4*, *m6*, *mα*); *m1 *which encodes a serine protease inhibitor and l(3) *groucho (gro) *which encodes a co-repressor of the E(spl) bHLH protein family. Although the E(spl) bHLH proteins are partly redundant and, therefore, a loss or addition of genes could be without consequences, the complex is highly conserved in all studied Drosophilids with respect to gene order and number, transcription orientation and overall size. As expected, the evolution rate of the orthologs is different. The best conservation is found between the Gro orthologs (more than 96% identity, Fig. 1), whereas M1 displays the highest evolutionary rate (less than 61% identity between *D. melanogaster *and *D. virilis*; Fig. 1).

### The bHLH proteins of the *E(spl)-C*

In the Drosophilids, all *E(spl) *bHLH genes are without intron, and the proteins contain the typical bHLH and orange domains and end with the WRPW motif. The best conserved bHLH ortholog is Mγ followed by M3, Mβ, M5, Mδ and M7/8 (Fig. 1). However, the evolutionary rate varies quite strongly. Comparing *D. melanogaster *with *D. virilis*, the highest identity score is found for Mγ with ~92% (Fig. 1A,B) and the lowest for M8 with ~81%. An even more striking difference in the identity scores is observed when comparing the *D. melanogaster *and *D. pseudoobscura *orthologs M3 and M7 (93% versus 73%; Fig. 1A,C). The low degree of conservation of the M7 proteins is rather surprising. It is based on one hand on peculiar size variations: 206 residues in *D. pseudoobscura*, 186 in *D. melanogaster *and 197 in *D. virilis *(Fig. 1C). On the other hand, the amino acid composition of the bHLH and orange domains is much better conserved between *D. melanogaster *and *D. virilis *than between *D. melanogaster *and *D. pseudoobscura*. This is different from all the other bHLH orthologs: the bHLH domains of M3 and Mβ are identical in all three species and also the other ones are extremely similar. Only one conservative change is detected in the Mγ bHLH domain of *D. melanogaster *compared with *D. virilis*, and just two in M5. The bHLH domains of the M8 and Mδ proteins contain also single non-conservative replacements, apart from a few conservative changes. However, *D. pseudoobscura *M7 shows an unusual high number of changes – six replacements and five conservative changes – within the bHLH domain if compared to the *melanogaster *ortholog (Fig. 1C).

### The Bearded-protein family in the *E(spl)-C*

In general, the Brd-type proteins evolve faster than the bHLH proteins: M4 and Mα are the best conserved members with ~82% identity between *virilis *and *melanogaster *and are thus within the range of the fastest evolving *m7/8 *bHLH coding genes (Fig. 1). The overall structure of M4 and Mα orthologs is quite similar in all studied *Drosophila *species. The so-called 'bearded'-domain is completely identical in the Mα orthologs, M4 has only a few gaps. However, the predicted *D. virilis m4 *gene has an extended open reading frame of novel 132 residues at the 5' end, whereas the remaining 156 residues are conserved. The *melanogaster m4 *5' region reveals similarity at the DNA level, however, has no open reading frame. Therefore it remains questionable whether the larger open reading frame in *D. virilis *is indeed translated. The other two Brd-family members, M2 and M6, are much less conserved: Only approximately 68% identity is found between the respective orthologs of *D. melanogaster *and *D. virilis*. Despite this little conservation, the typical Brd protein domains can still be recognized. The most prominent is the predicted basic amphipathic α-helix domain in the N-terminal protein region [14].

### The *m1 *gene in the *E(spl)-C*

The *m1 *gene encodes a protein that has the signatures of serine protease inhibitors [15]. Despite a low degree of conservation which ranges between 60 and 70% identity between the M1 orthologs (Fig. 1A), the structurally important cysteines residues are conserved in number and spacing [15]. Notably, the orthologous genes of *D. pseudoobscura *and *D. virilis *have a significantly longer open reading frame at the 5'end that extends the proteins for approximately 50 residues to 203 in the case of *D. virilis*. The extended protein parts share ~68% similarities within the first 30 residues between the two orthologs. Furthermore, the first nine residues have only one conservative exchange arguing for its translation in vivo. In *melanogaster *all three reading frames at the 5' end are blocked by several stop codons, excluding a likewise 5' extension. However, at the DNA level there are identities of 69% to the *virilis *ortholog and 79% to the *pseudoobscura *ortholog which could be also interpreted as conserved regulatory sequence.

### The *E(spl)-C *in *Anopheles gambiae*

**Figure 2:**
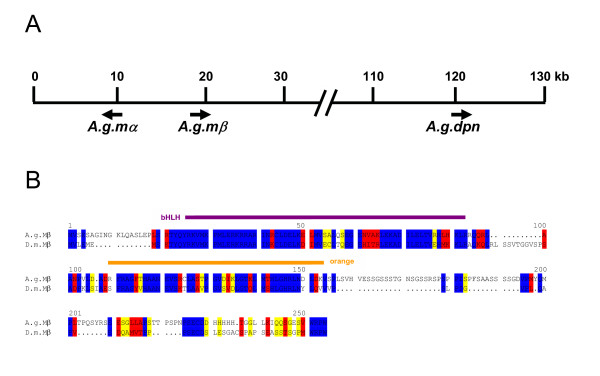
**The *E(spl)-C *in mosquito *A. gambiae***. (A) The *E(spl)-C *in the mosquito is composed of two putative genes, *A.g.mβ *and *A.g.mα*. Approximately 100 kb away a second bHLH coding gene was detected, however, the analysis predicted a close relationship to Deadpan and Hairy. Since a good fitting *hairy *ortholog is elsewhere in the mosquito genome, the gene is most likely a *dpn *ortholog. B) Alignment between A.g.Mβ and D.m.Mβ shows good conservation within the bHLH and the orange domains (marked) as well as the WRPW motif at the C-terminus. Identical residues are marked in blue; red shows highly related and yellow similar residues.

**Figure 3:**
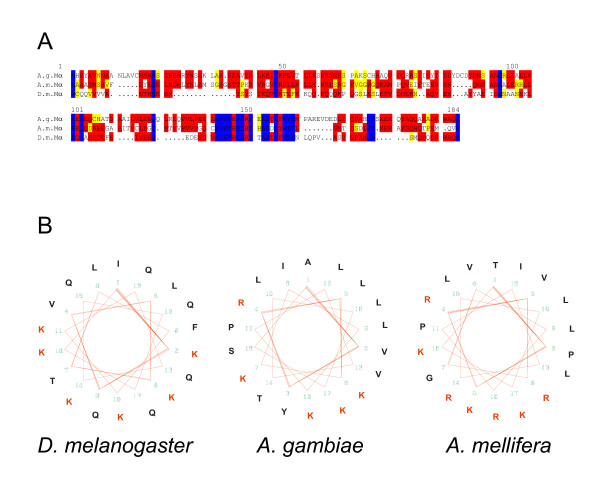
**Conservation of the Brd-family member Mα**. A) Alignment of the presumptive Mα proteins of *D. melanogaster*, *A. mellifera *and *A. gambiae*. Although the alignment reveals not much identity (blue), the postulated features that typify Brd-proteins are present [14]. Red, highly related and yellow, similar residues. B) Mα contains a typcial amphipathic α-helix with high concentration of lysine (K) and arginine (R) residues on one side of the wheel (red).

Albeit *Anopheles *belongs to the dipteran flies it does not contain an *E(spl)-C *that matches that of Drosophilids (Fig. 2A). Only a single transcription unit with respectable conservation that contains one intron was annotated in the genome project (ENSANGG00000017601; see Tab. 1). However, the predicted coding sequence does not end with a WRPW motif as expected for E(spl) bHLH proteins. By searching through the genomic sequence, we propose a different gene structure, where the transcript extends into the intron that maintains an open reading frame and ends with a WRPW motif. In this case, the *E(spl) *homolog of mosquito would be without intron and shares highest similarity to the Mβ/Mγ pair of *D. melanogaster *(80.3/75.7% similarity and 69.6/67.4% identity, respectively). Based on the similarity, we propose that it corresponds to *D.m.mβ *(Fig. 2B) and named it therefore *A.g.mβ*. Moreover, there is a single Brd-like gene in close proximity (~8 kb) of *A.g.mβ *(Fig. 2A). This gene encodes a protein most similar to the *D. melanogaster *Mα protein with almost 60% identity (Fig. 3A); therefore we named it *A.g.mα*. It shares all features of the Brd-family proteins described earlier [14], including the amphipathic α-helix domain in the N-terminal part (Fig. 3B).

**Table 1:** Identity matrix of E(spl) bHLH proteins in *D. melanogaster *(% similarity/identity)

	Mβ	Mδ	M8	M7	M5	M3	Ø
Mγ	75/68	68/59	69/57	75/67	72/61	74/64	72/63
Mβ		67/56	70/61	75/68	71/60	80/71	73/64
Mδ			64/50	67/59	65/50	67/53	66/55
M8				67/59	80/73	63/50	69/58
M7					67/61	70/61	70/63
M5						66/57	70/60
M3							70/59

Ø = average similarity/identity (%) of one bHLH member if compared to all others

Approximately 100 kb from the 3' end of *A.g.mβ*, we detected another sequence that might encode an E(spl) bHLH type protein (Fig. 2A; ENSANGG00000017548). However, the presumptive gene product is more highly related to *D. melanogaster *Deadpan (Dpn; 57% identity) than to E(spl) bHLH Mβ (52% identity). The conservation extends beyond the amino acid sequence: we find the same intron/exon structure in this *Anopheles *gene as in the *deadpan *and *hairy *genes from *Drosophila *(see also Fig. 8a). We believe that this *Anopheles *protein corresponds to Dpn (A.g.Dpn) rather than to Hairy, since it shares little more than 47% identity with *D. melanogaster *Hairy. The best hit with the *D. melanogaster *Hairy protein is found on the second chromosome in *Anopheles *(*A.g.h*; 72% identity at protein level). Other genes of the *Drosophila E(spl)-C *were not detected nearby: maybe they are not conserved enough to be discovered like e.g. *m6 *or they are located at totally different positions in the genome like *gro*.

### The *E(spl)-C *in *Apis mellifera*

**Figure 4:**
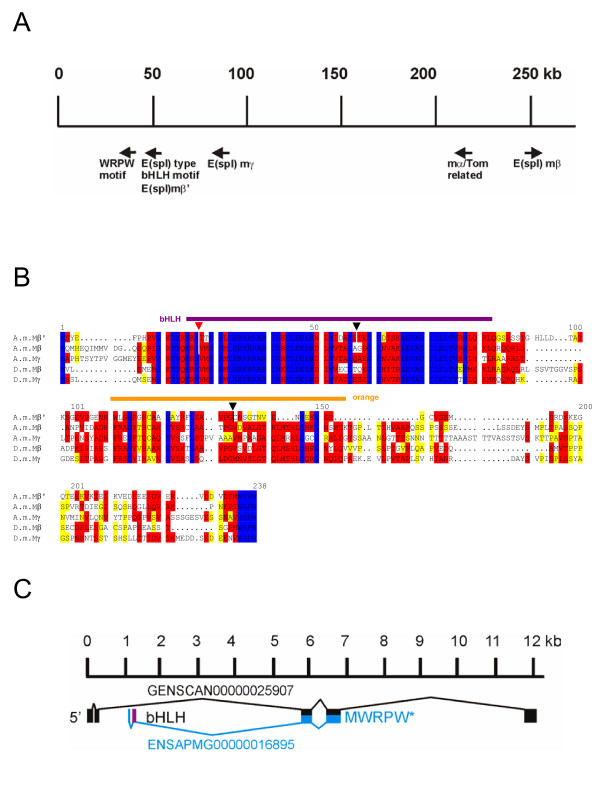
**The *E(spl)-C *in *Apis mellifera***. A) A genomic region spanning about 250 kb (GroupUn.159; numbers are correspondingly) contains 3 presumptive *E(spl) *bHLH genes and one *mα *related gene. The gene at position ~250 kb encodes a bHLH protein with best overall identity to D.m.Mβ. This gene is disrupted by an intron inside the bHLH domain (dash and red arrowhead in B). Close by, at position 220 kb a *mα *related coding region is found. Based on high similarity to D.m.Mα and its close neighbourhood to *A.m.mβ*, we named it *A.m.mα*. A second intronless *E(spl) *like bHLH coding gene is located at position ~90 kb. We name it *A.m.mγ *since the encoded protein shows best overall identity to the D.m.Mγ. Approximately 50 kb away we detected sequences encoding an E(spl) like bHLH domain (A.m.Mβ') and a long open reading frame ending with a WRPW motif. The Ensembl honeybee database annotated a gene with five introns spanning 12 kb within this region but missed the respective motifs (GENSCAN00000025907). We propose a different gene structure; see Figure C for details. B) Alignment of the three putative honeybee bHLH proteins (A.m.Mβ', A.m.Mβ, A.m.Mγ) with *D. melanogaster *Mβ and Mγ proteins is shown. Identical residues are marked in blue; red shows highly related and yellow similar residues. Intron positions are marked with a triangle above and a dash in the respective *A. mellifera *sequence. C) Structure of the 12 kb GENSCAN00000025907 region. Black shows the Ensembl gene annotation, and blue the new ENSAPMG00000016895 annotation. Purple highlights a second exon that encodes part of the bHLH domain, the open reading frame extends into the predicted intron of GENSCAN00000025907 and terminates with WRPW.

Like in the mosquito, there is no extended *E(spl)-C *in the honeybee. In fact, we find a similar structure of one E(spl) bHLH type and one Brd-type gene that share highest homology to Mβ and to Mα, respectively (Fig. 4A). The relative transcription orientation (head to head) is the same, suggesting that *mβ *and *mα *represent the ur-complex (compare Figs. 1A, 2A, 4A). However, the situation in honeybee is more complicated in several respects.

For example, the *A.m.mα *homolog is predicted to consist of five exons that code for a protein with 402 residues. This is considerably larger than the 138 residues of D.m.Mα. Again, we propose a different gene structure based on the analysis of the genomic DNA, where the translation extends into the first postulated intron and ends shortly afterwards. The encoded protein than consists of only 168 amino acids and terminates with residues that are very similar to the Mα *Drosophila *homolog (M Q V A) (Fig. 3A). Moreover, it shows the typical amphipathic α-helix domain in the N-terminal part (Fig. 3B). The other motifs are also conserved, the extreme C-terminus is, however, only similar (Fig. 3A). This protein shows also high similarity to *Drosophila *Twin of m4 (Tom) that might slightly exceed that to D.m.Mα depending on the parameters used. As in mosquito, no other Brd-like protein was found in the honeybee database such that a Brd-complex seems non-existent, unlike in Drosophilids [33].

The *Apis *database annotates a single intron for the *A.m.mβ *gene that conforms to the GT AG rule. This is in contrast to all the *Drosophila E(spl) *bHLH genes that are intronless. Moreover, there are several possible start sites and it remains unclear which one is used. In *D. melanogaster*, there are other bHLH-WRPW encoding genes that contain introns, like *deadpan *(*dpn*) or *Side*. However, the respective protein sequences do not align well with A.m.Mβ, and the *Apis *genome contains predicted orthologs to these genes at other locations (see below). The A.m.Mβ protein is also highly similar to *melanogaster *Mγ with 72.3 versus 77.7% similarity and 65 versus 67.5% identity compared to D.m.Mβ (Fig. 4B). These differences are extremely small. In fact, the *Apis *database proposes this gene as *mγ *based on the similarity within the bHLH domain. However, we used for our comparison the entire protein sequences and calculated identity overall. Moreover, based on the *E(spl)-C *structure in *Anopheles*, we favour the hypothesis that *mβ *is the ancestral gene.

Within the ~250 kb contig (GroupUn. 159), there are two further stretches that might encode E(spl) type bHLH proteins. One is located about 150 kb apart. This gene contains no introns and the encoded protein shows homology to both Mβ and Mγ. In this case, the similarity seems slightly higher to Mγ than to Mβ (72.2 vs. 69.3% similarity and 66 vs. 62.7% identity). Therefore, we call the gene *A.m.mγ *(Fig. 4A,B). Both FlyBase and BlastN give a higher score to *D. melanogaster *Mβ than to Mγ. Presumably, both use similar paradigms based on an alignment of only the best conserved sequences, whereas we used less stringent parameters (see Methods) to allow an alignment of the complete sequences. As to be expected, the two E(spl) honeybee proteins A.m.Mβ and A.m.Mγ are highly related to each other with a similarity of 73% and an identity of 67%. Interestingly, this numbers are very similar to those from a likewise comparison of the *D. melanogaster *Mβ and Mγ homologs (Table 1).

Another 50 kb further up at position 50 (Fig. 4A), we found a short alignment to the E(spl) bHLH domain. However, there was no predicted gene, nor a start codon, nor a WRPW motif. Nearby at position ~46 kb there is an open reading frame of 133 residues split by one intron that belongs to a predicted database gene consisting of five exons (GENSCAN00000025907; black in Fig. 4C). However, lacking any similarity to known genes of *Apis *or other species, this gene remained without functional prediction in the *Apis *database. In agreement, our searches in the FlyBase did not detect any similar sequences. However, we predict an E(spl) bHLH-type protein encoded by this gene region: extension of the open reading frame into the adjacent intron ends in WRPW (Fig. 4C, blue exons). The bHLH encoding sequences (purple in Fig. 4C) are located within the second predicted intron of the putative gene shown in black. There are respective exon/intron boundary consensus sequences to allow for a single transcript that contains the bHLH domain, an orange domain and ends with the WRPW motif (Fig. 4C). This third *E(spl) *bHLH gene would comprise five exons. In fact, in the update May 2005 of the Ensembl honeybee database, the Ensembl automatic analysis pipeline predicts a very similar protein, however, with a different N-terminus and slightly smaller third and fourth exons. This gene than consists of four exons which would be similar to *D. melanogaster hairy *and *dpn *that contain two introns. However, the encoded protein is most similar to the E(spl) protein Mβ, so we call the gene *A.m.mβ'*. Since Apis *hairy *and *dpn *are found elsewhere in the genome (see below), we propose that the *E(spl)-C *in *Apis mellifera *consists of the ur-complex plus two further *E(spl) *bHLH genes most closely related to *mγ *and *mβ*. No other genes of the *Drosophila E(spl)-C *are present in that of the honeybee. We find a highly conserved Groucho ortholog, however, at a completely different position in the genome.

### Conservation of other Hairy/E(spl)-like proteins known from *Drosophila*

In total, 12 genes are known in *D. melanogaster *to encode Hairy/E(spl)-like proteins, i.e. bHLH proteins that also have the orange domain and a WRPW-type Gro-binding motif (see Table 2). Apart from the seven E(spl) bHLH proteins, these include Hairy, Deadpan, Side, Hey and Her [34]. Moreover, there is similarity to Stich1/Sticky which has a bHLH and an orange domain but not the typical Gro-binding motif [35]. Since the number of *E(spl) *bHLH genes is not conserved in honeybee and mosquito, it was interesting to ask whether all the other genes were present. We searched the Ensembl database with the respective *D. melanogaster *protein sequences and found orthologs of all genes except of *Her *in both species (see Table 2). However, most of the predictions are incomplete. We know from *D. melanogaster *that these genes contain introns, which complicates the search for potential coding sequences within genomic DNA. Thus, our protein sequence predictions are uncertain. With the sole exception of Dpn, all the proteins are better conserved between *Drosophila *and *Anopheles *than between *Drosophila *and *Apis*, confirming the evolutionary relationship. The best conserved proteins are Hey and Hairy. The Hey orthologs are 76% identical between *Drosophila *and *Anopheles *and 66% between *Drosophila *and *Apis *and the Hairy orthologs between 72% and 65%, respectively. Less conservation is found for Side, Dpn and Stich1 (62/57 Side, 57/59 Dpn and 60/57 Stich1; % identity comparing fly with mosquito and honeybee, respectively). All proteins share the bHLH and orange domains. The WRPW motif of Hairy, Dpn and Side as well as the YRPW motif of Hey is present in the orthologs.

**Table 2:** Gene annotation used by the respective databases

***D. melanogaster***	***D. pseudoobscura *** (contig 4374 Contig 4847)	***D. virilis***	***Apis mellifera***	***Anopheles gambiae***
*D.m. mδ *(CG8328)	*D.p. mδ *(178 652-178 089)	*D.v. mδ*	*-*	*-*
*D.m. mγ *(CG8333)	*D.p. mγ *(175 594-174 953)	*D.v. mγ*	*A.m. mγ *(ENSAPMG0000004887)	-
*D.m. mβ *(CG14548)	*D.p. mβ *(169 335–169 934)	*D.v. mβ*	*A.m. mβ *(ENSAPMG0000004881)	*A.g. mβ *(ENSANGG00000017601)
*D.m. mα *(CG8337)	*D.p. mα *(163 786-163 358)	*D.v. mα*	*A.m. mα *(GENSCAN0000001764)	*A.g. mα *(SNA00000011401)
*D.m. m1 *(CG8342)	*D.p. m1 *(158 207–158 734)	*D.v. m1*	-	-
*D.m. m2 *(CG6104)	*D.p. m2 *(157 012–157 407)	*D.v. m2*	-	-
*D.m. m3 *(CG8346)	*D.p. m3 *(151 792-151 136)	*D.v. m3*	-	-
*D.m. m4 *(CG6099)	*D.p. m4 *(149 049–149 516)	*D.v. m4*	-	-
*D.m. m5 *(CG6096)	*D.p. m5 *(142 321–142 881)	*D.v. m5*	-	-
*D.m. m6 *(CG8354)	*D.p. m6 *(137 883-137 656)	*D.v. m6*	-	-
*D.m. m7 *(CG8361)	*D.p. m7 *(134 008-133 391)	*D.v. m7*	-	-
*D.m. m8 *(CG8365)	*D.p. m8 *(130 185-129 628)	*D.v. m8*	-	-
*D.m. gro *(CG8384)	*D.p.gro *(148 473-117 489)	*D.v. gro*	-	-
			*A.m. mβ' *(ENSAPMG00000016895)	
*D.m. stich1 *(CG17100)	not analysed	not analysed	*A.m. stich1 *(ENSAPMG00000005857)	*A.g. stich1 *(ENSANGG00000016365)
*D.m. side *(CG10446)	not analysed	not analysed	*A.m. side *(ENSAPMG0000000088)	*A.g. side *(ENSANGG00000014329)
*D.m. dpn *(CG8704)	not analysed	not analysed	*A.m. dpn *(ENSAPMG00000004551)	*A.g. dpn *(ENSANGG00000017548)
*D.m. Hey *(CG11194)	not analysed	not analysed	*A.m. Hey *(ENSAPMG0000000726)	*A.g. Hey *(ENSANGG00000021744)
*D.m. Her *(CG5927)	not analysed	not analysed	-	-
*D.m. h *(CG6494)	not analysed	not analysed	*A.m. h *(ENSAPMG00000004545)	*A.g. h *(ENSANGG00000018369)
				
*D.m. ac *(CG3796)	not analysed	*D.v. ac*	-	-
*D.m. sc *(CG3827)	not analysed	*D.v. sc*	-	-
*D.m. l'sc *(CG3839)	not analysed	*D.v. l'sc*	*A.m. ash *(ENSAPMG00000003261)	*A.g. ash *(ENSANGG00000010650(Q95VY6)
*D.m. ase *(CG3258)	not analysed	*D.v. ase*	*A.m. ase *(ENSAPMG00000003265)	*A.g. ase *(ENSANGG00000015341)
				
*D.m. da *(CG5102)	not analysed	not analysed	*A.m. da *(ENSAPMP00000005673)	*A.g. da *(ENSANGEST00000361691/SNAP000000012539)
*D.m. Ocho *(CG5138)	not analysed	not analysed	-	-
*D.m. Tom *(CG5185)	not analysed	not analysed	-	-
*D.m. Brd *(CG3096)	not analysed	not analysed	-	-

(-, not found)

### The *Achaete-Scute complex *in Drosophilids

**Figure 5:**
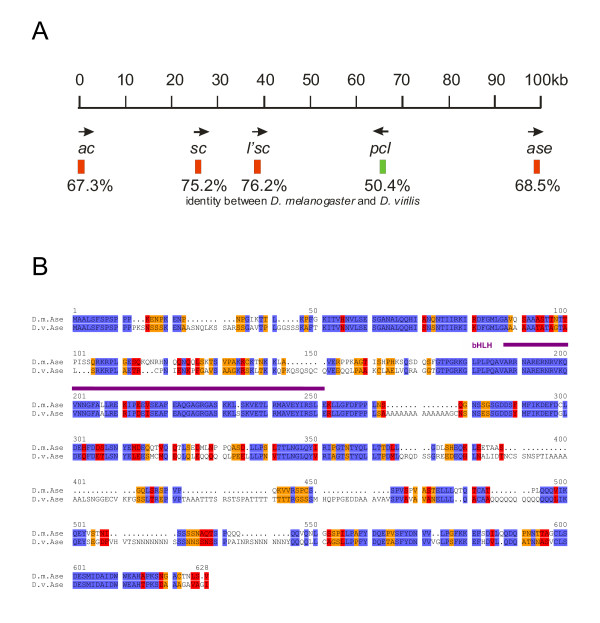
**The *AS-C *of *D. virilis***. A) The *AS-C *is highly conserved between *D. melanogaster *and *D. virilis *concerning gene number, transcript orientation and overall size. At the protein level (% identity) the best conservation is found between the Sc and L'sc orthologs. However, the conservation rate is lower compared with the E(spl) bHLH proteins (see Fig. 1). The *pcl *gene, although only 50.4% identical, is found between *l'sc *and *ase*. Because of gaps in the genomic *virilis *sequence, a scheme of the *D. melanogaster *complex is shown. B) Alignment of the Ase protein orthologs of *D. melanogaster *and *D. virilis*. Note the extension of the D.v.Ase protein by repetitive amino acid stretches composed of poly N, poly Q and poly A. The best conservation is found within the bHLH domain (purple). Identical residues are marked in blue; red shows highly related and yellow similar residues.

The *Achaete-Scute complex (AS-C) *is well conserved in *D. virilis*: all four genes, *achaete (ac)*, *lethal of scute (l'sc)*, *scute (sc) *and *asense (ase) *are found in the same order and orientation on the X-chromosome (Fig. 5A). Like in *D. melanogaster*, the genes are without introns. All proteins share the typical bHLH motif of the *AS-C *proteins and this domain reveals the lowest evolutionary rate. However, compared with the bHLH proteins of the *E(spl)-C *the bHLH proteins of the *AS-C *evolve faster. The complex can be separated into two clusters that are distinguished by their rate of conservation. On one hand, L'sc and Sc are well conserved with an identity between *D. melanogaster *and *D. virilis *of more than 75% and on the other hand Ac and Ase with an identity of less than 69% (Fig. 5A). Note that the highest divergence that was found between these two species in the *E(spl)-C *was for M8 with still almost 81% identity.

Of the four *AS-C *gene members in *D. melanogaster*, *ase *stands out because it is much larger than the other three. In *D. virilis*, the size increase is even more striking: D.v.Ase is predicted to comprise 619 residues, whereas D.m.Ase is only 486 residues in length (Fig. 5B). This extension of more than 20% additional residues is caused by multiple insertions of repetitive sequences that code for poly-glutamine (Q), poly-alanine (A) and poly-asparagine (N) stretches (Fig. 5B). Like in *D. melanogaster *the unrelated gene *pepsinogen-like (pcl) *is located between *l'sc *and *ase *(Fig. 5A).

### The *AS-C *in *Anopheles gambiae*

**Figure 6:**
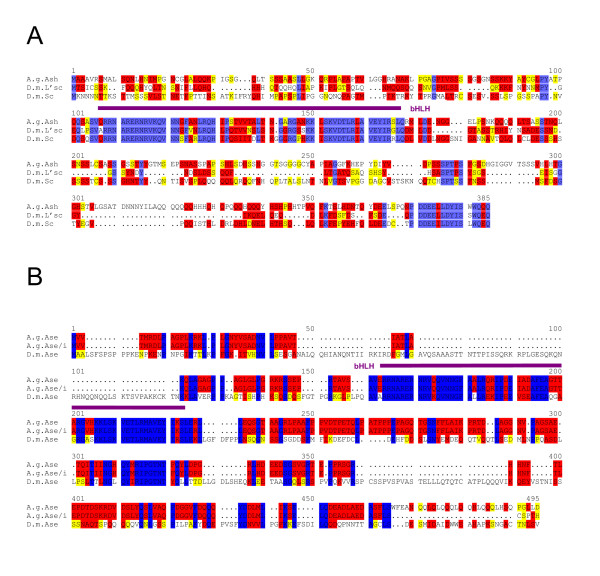
**The *AS-C *in *A. gambiae***. A) Alignment of the *achaete-scute *homologous protein of *A. gambiae *(A.g.Ash; [36]) with *melanogaster *L'sc and Sc proteins. Note the high conservation of the bHLH domain (purple) and the very C-terminus. Comparison over the entire length gives a higher identity score to D.m.L'sc, however, the alignment shows also regions that are more similar to D.m.Sc. B) Alignment of the neighbouring bHLH gene product from *A. gambiae *with *D. melanogaster *Ase. The alignment is shown to A.g.Ase/i (database predicted version without intron) and A.g.Ase (second intron translated). The second predicted intron comprises almost 2.5 kb and ends with an exon translated into five residues (CSPTH; in A.g.Ase/i). Translation into this intron leads to A.g.Ase that is similar in size and in its terminus to D.m.Ase. Highest conservation is found in the bHLH domain (purple). Identical residues are marked in blue; red shows highly related and yellow similar residues.

In the mosquito, we find only two potential *achaete-scute *like genes that are in close neighbourhood of less than 30 kb. Interestingly, like in *Drosophila *they are located on the X-chromosome. One of them encodes a protein that is very similar to L'sc and Sc proteins not only within the bHLH domain but also at the C-terminus (Fig. 6A). This is not unexpected because the C-terminus is involved in transcriptional activation as well binding of E(spl) bHLH proteins [36]. The BestFit program gives a higher score to L'sc (64% identity) than to Sc (57% identity). However, closer inspection reveals that some protein regions are more similar to D.m.Sc and others more to D.m.L'sc (Fig. 6A) suggesting common ancestry for this gene pair. The *Anopheles *data base predicts an intron, which however retains the open reading frame. In fact, Wülbeck and Simpson [37] cloned and sequenced the respective *A.g.ash *cDNA and showed that it is intronless. We detected three conservative amino acid exchanges between the published A.g.Ash protein sequence and that obtained from translating the database genomic DNA, namely at position 8 (M-L), position 189 (T-S) and position 311 (Q-H).

The second presumptive gene has two predicted introns. The derived amino acid sequence shares between 54% and 57% identity with all four *D. melanogaster AS-C *proteins. By searching the *D. melanogaster *genome with the predicted protein sequence, the best hit was found to Ase protein (Fig. 6B). Accordingly, the Anopheles database defined it as *ase *homolog and so we named it *A.g.ase*. We note, however, that a precise appointment is difficult based on the lack of a significant similarity at the C-terminus which normally allows the distinction between the *AS-C *members.

### The *AS-C *in honeybee

**Figure 7:**
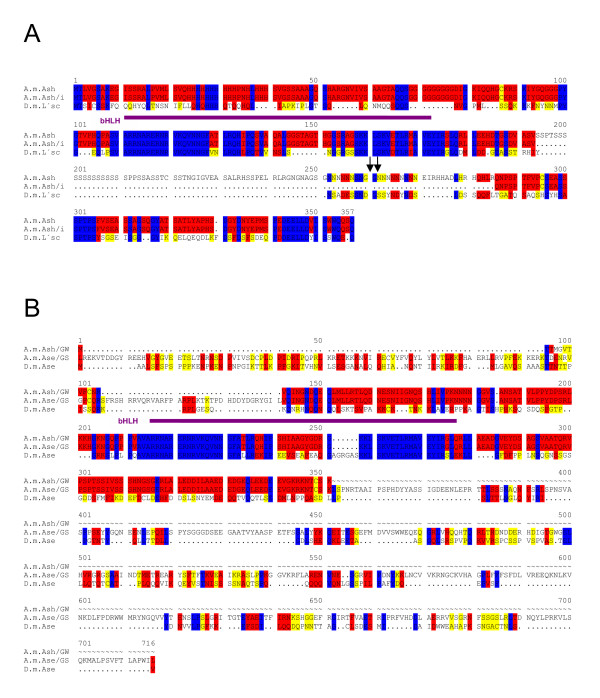
**The *AS-C *in *A. mellifera***. A) Comparison of D.m.L'sc with the predicted A.m.Ash protein. Two forms were compared, without intron sequence, A.m.Ash/i, and with translated intron, A.m.Ash. Arrows mark additional splice consensus sites. B) Within 40 kb of *A.m.ash*, there is a second potential gene encoding a widely diverged bHLH protein. Two different programs were used for gene prediction that gives A.m.Ase/GS (Chris Burge's Genscan program) and A.m.Ase/GW (GeneWise model); both predicted proteins were aligned with D.m.Ase. Decent conservation is only found in the putative bHLH domains (purple). Identical residues are marked in blue; red shows highly related and yellow similar residues.

Like in the mosquito, the honeybee genome encodes only two *AS-C *like proteins. The two transcription units are the predicted Ensembl genes ENSAPMG00000003261 and ENSAPMG00000003265 (Table 2). They are located in the scaffold group 10.3 and are approximately 40 kb apart from each other. The former encodes a protein that is highly similar to the L'sc/Sc protein pair, so we named it *A.m.ash *(Fig. 7A). In contrast to the *Drosophila AS-C *genes, *A.m.ash *is predicted to contain a single intron which however, retains an open reading frame. Therefore, like in mosquito the encoded protein could be significantly larger. We thus aligned the protein sequences of A.m.Ash with and without translation of the predicted intron with D.m.L'sc (Fig. 7A). Since the open reading frame of the presumptive intron is translated primarily into serine residues, no alignment with the D.m.L'sc protein was possible for this part, supporting the intron prediction. We note that there are two more exon/intron boundary consensus sequences within the predicted intron (arrows in Fig. 7A). If these were used instead of the ones predicted, the intron would be somewhat smaller and A.m.Ash accordingly 25 amino acids larger. As shown in Fig. 7A, the resultant protein would be more similar in size to *melanogaster *L'sc protein and moreover, share additional similarities in this part of the protein. The *Apis *database does not provide a start for A.m.Ash, which we could deduce however from the alignment with the *D. melanogaster *protein.

**Figure 8:**
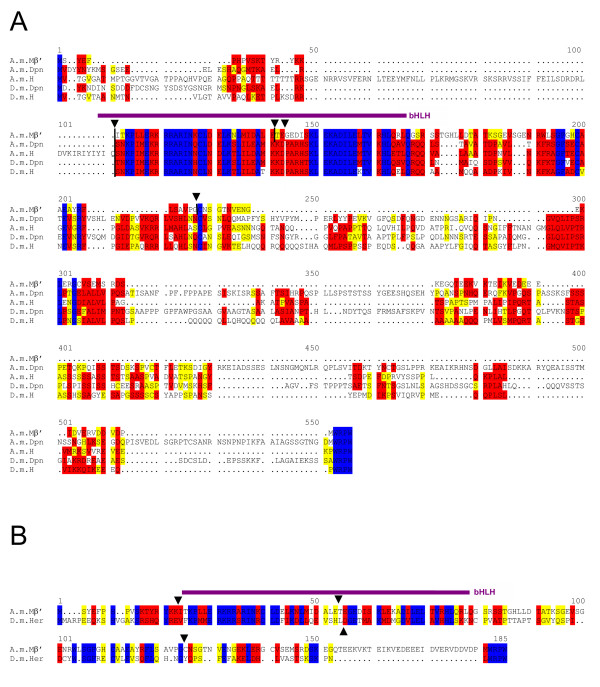
**Comparison of A.m.Mβ' with Dpn, Hairy and Her proteins**. A) Comparison of A.m.Mβ' with A.m.Dpn, A.m.H, D.m.Dpn and D.m.H. The A.m.Mβ' protein belongs to the *E(spl)-C*, however, has several introns (triangle on top, vertical dashes within sequence). Two of the introns are within the bHLH domain at similar position as in *D. melanogaster hairy *or *dpn *genes. The bHLH domain is indicated in purple. B) Alignment of *D. melanogaster *er Hwith A m.Mβ' protein. *Her *has only one intron, however, at very similar position as the second intron of *A.m.Mβ'*. Introns are marked with triangles.

Interpretation of the second gene is much more difficult. The annotation by Ensembl automatic pipeline using GeneWise model based on either protein or aligned EST's resulted in a coding sequence within a single exon that lacked both start and stop codons, however, aligned well with *AS-C *bHLH domains. We thus extended our studies into the surrounding genomic DNA, where we detected one further open reading frame. The deduced amino acid sequence matched well with the N-terminal part of *D. melanogaster *Ac and Ase proteins. However, the C-terminus did not align convincingly (Fig. 7B/GW). Another gene prediction using Chris Burge's Genscan program [38] gave a transcript of 8 exons spanning over 8 kb of genomic DNA. The translation gave a larger protein again without start methionine that contained the single exon predicted by the GenWise model (Fig. 7B/GS). Again there was very little similarity in the C-terminal part of this and *Drosophila AS-C *proteins (Fig 7B). Because Genscan could not predict the protein start, we propose a combination of both models with the N-terminus as shown in the A.m.Ase/GW sequence and the C-terminus as in A.m.Ase/GS that might, however, end shorter than shown in Fig. 7B.

The high divergence from the *Drosophila AS-C *proteins renders precise predictions very difficult. In fact, under standard conditions like FlyBase BlastN only parts of the bHLH domain can be identified. Comparison with the *D. melanogaster AS-C *proteins gave minimally different scores, with the highest score found with D.m.Ac followed by D.m.Ase, dependent on the parameters. For example, the *Apis *database finds best scores with *melanogaster *Sc and L'sc. We named this gene *A.m.ase *by its similarity notably in the N-terminal part and based on the arrangement of the mosquito *AS-C*.

### Conservation of predicted regulatory elements in *E(spl)-C *and *AS-C*

Neurogenesis in *Drosophila *is subjected to various levels of regulation. As described in the introduction, E(spl) bHLH proteins repress proneural gene activity. Negative regulation is brought about by repression of transcription as well as at the protein level [36,39-42]. A further mode of regulation involves RNA:RNA duplexes [43]. These are formed by small sequence stretches located in the 3' untranslated region (3' UTR) of the mRNAs of proneural *AS-C *genes and different members of the *E(spl)-C*. For example, *l'sc *mRNA contains so-called proneural boxes (PB-box) (GGAAGAC) which bind to the GY-boxes (GUCUUCC) of *E(spl) *m4 RNA [43]. We searched the genomic sequences adjacent to the coding sequences for respective regulatory elements and found them in *D. virilis*: there are two PB boxes in *D.v.l'sc *and GY-boxes in the 3'UTR of the predicted *virilis E(spl) *genes *m3*, *m4*, *m5 *and *mγ *just as in *D. melanogaster *and meanwhile published by Lai et al. [33]. Moreover, it has been shown that *E(spl) *genes are direct targets of the Notch signal involving the DNA binding protein Su(H) [11,13,15,19]. Accordingly, there are potential Su(H) binding sites (C/TGTGA/GGA) in all *D. melanogaster E(spl) *genes including *m2 *and *m6 *[14,15], which are also present in the respective *virilis *orthologs [44]. However, the predicted binding sites for proneural bHLH activators (E box: GCAGGTG) [14] are less well conserved during evolution. Whereas the *D. virilis m2 *and *m4 *orthologs contain such a regulatory element, we found no sequence fitting the E-box consensus in either the *D.v.m6 *or *D.v.mα *sequence.

In mosquito and honeybee, regulatory elements of RNA:RNA duplex-type were not detected. None of the two *AS-C *genes of either *Apis *or *Anopheles *contained PB-box like sequences in the 3' UTR, albeit the highly diverged sequence and gene structure of *A.g.ase *does not allow to definitely exclude their presence. Since there is no predicted *m4 *ortholog in *Anopheles *and *Apis*, we looked at the 3' end of the *mα *gene as the single Brd-family member. However, in none of these gene sequences did we find GY type boxes like in *Drosophila*. We note that some of the predicted gene structures are still incomplete and a search for small sequence stretches is notoriously difficult if one allows for variations. Therefore, there is the formal possibility that we have missed these sites.

## Discussion

### The *Enhancer of split *complex

Extensive genome analyses in the recent years revealed that there are not many examples of large gene complexes that are widely conserved. Prominent examples are the HOX (homeobox) complexes, which contain homeotic genes in *Drosophila*. HOX complexes are well conserved in metazoans despite some variations in gene number. HOX-genes encode regulatory proteins with specific individual functions and mutations affect different aspects of the body plan [45]. Not surprisingly, it is almost only the homeodomain, which serves as sequence-specific DNA binding motif that is conserved amongst different species [46]. In contrast, similarity amongst bHLH proteins encoded by the *E(spl)-C *extends over the entire length, even within the same species indicating rather recent duplication events. The *D. melanogaster *proteins M8/M5 and Mβ/M3 are most similar with over 70% identity, whereas Mδ is the most diverged. However, Mδ still shares at least 50% identity with other *E(spl) *bHLH protein members (see pair wise comparison in the identity matrix of Mδ with M8, M5 and M3; Table 1). More interesting is the analysis of the overall similarity amongst these proteins. Here, any one of the proteins is compared with the other six and the result is averaged. Clearly, Mβ (73/64%, similarity/identity) closely followed by Mγ (72/63%) is most similar to all others, whereas Mδ (66/55%) shows the lowest values (Table 1). One interpretation might be that the different bHLH genes evolved by duplication out of *mβ *or *mγ*. Remarkably, these two bHLH proteins besides M3 are the best conserved in the three *Drosophila *species (Fig. 1A). We would like to postulate that these are the most ancient proteins with the most general function and, therefore, the highest selection pressure. This hypothesis is supported by the finding that *mβ *has the most general expression pattern from which the others can be derived by a decrease of gene activity [19]. The conspicuous conservation of M3 might hint to an important function during egg development as this gene is expressed also maternally [17,22]. The high degree of conservation of all *E(spl) *bHLH orthologous proteins in Drosophilids, which is clearly higher than the similarity within this protein family in *D. melanogaster*, indicates specific and non-redundant roles during development (see also [23]). Some of these functions have been identified in the past [19,39]. It is conceivable that regulatory sequences were not duplicated or evolved more rapidly so that we now find highly dynamic expression patterns of these genes.

### The ancestral *E(spl)-C *is composed of *mβ *and *mα*

As outlined above, *mβ *appears to be the ancestral bHLH gene of the *E(spl)-C *in Drosophilids based on its great similarity with all the other bHLH proteins. This assumption is strongly supported by the sequence conservation of the E(spl) bHLH proteins in *A. gambiae *and *A. mellifera*. The single E(spl) bHLH protein encoded by the mosquito genome has the highest identity to Mβ. The genome of honeybee contains three prospective genes that encode proteins most highly related to E(spl) D.m.Mβ and D.m.Mγ. All three are clustered within a single sequence contig, albeit they span a large segment of about 250 kb, whereas the whole *E(spl)-C *in *D. melanogaster *comprises roughly 50 kb. Despite the fact that two of these genes possess introns just within the bHLH domain and at positions close to the ones found in the *D. melanogaster *genes *dpn*, *hairy *or *Her *(Figs. 4, 8), the amino acid sequence similarity classifies them clearly as E(spl) bHLH proteins. A comparison of *Anopheles *and *Apis *proteins reveals, that the presumptive Mβ homologs have highest similarity (83%) and identity (76%), whereas the protein that we classified as A.m.Mγ is just 70% similar and 66% identical to A.g.Mβ.

In Drosophilids, *mα *is located close to *mβ *and is transcribed in the opposite direction (head to head; Fig. 1A). This arrangement is likewise found in *Anopheles *and *Apis *(Figs. 2A, 4A). Notably, *A.m.mα *is next to *A.m.mβ*, whereas the two *Apis A.m.mγ *and *A.m.mβ' *genes are much further apart (Fig. 4). We find this arrangement to be very ancient. In the beetle *Tribolium*, which on the tree of evolution is found even more deeply rooted (~300 Myr to Dipterans [32]), two similar genes coding for Mβ-like proteins (~65% and ~67% identity to D.m.Mβ) are found and one is within ~18 kb to a gene coding for an Mα-like protein (~52% identity to D.m.Mα) (unpublished data derived from the Tribolium database). We postulate that the ur-complex consisted of these two ancestral genes, *mα *and *mβ*. It is intriguing that they belong to the two different classes of Notch-responsive genes in the *E(spl)-C*, the bHLH and the Brd-class. In the fly, *mα *and bHLH genes are similarly expressed [14,15,22]. It is not unlikely that they share common regulatory elements that could explain their co-segregation in the process of evolution.

What about the third bHLH coding gene found in Apis, *A.m.mβ'*? This gene may have derived by duplication of *A.m.mβ *or of *A.m.mγ*. It is peculiar that this protein is more similar to *D. melanogaster *Mβ protein than to either A.m.Mβ or A.g.Mβ (see Table 3). In contrast, A.m.Mγ is more similar to A.m.Mβ than to A.m.Mβ'. Furthermore this gene has three introns; one of them is larger than 4 kb reminiscent of *Drosophila hairy *or *dpn *intron sizes. We think that it is unlikely that *A.m.mβ' *encodes one of the other Hairy/E(spl)-type proteins since respective orthologs were found in the *A. mellifera *genome with the exception of *Her*, which seems also absent from the mosquito genome. However, there are similarities between the A.m.Mβ' and *D. melanogaster *Her proteins, including one intron which is at a similar position (Fig. 8B). Although highly speculative, one might conclude that the *Drosophila Her *gene originally derived from an ancient *E(spl) *bHLH type gene. However, this speculation has to be proved or disproved by further investigations. The fact that the positions of the introns of *Drosophila dpn *and *hairy *are identical and the introns in *A.m.mγ *and *A.m.mβ' *are at very similar positions (Fig. 8) supports the notion of a common ancestry of these genes.

**Table 3:** Comparison between *Apis mellifera*, *D. melanogaster *and *Anopheles gambia *(% similarity/identity)

	A.m.Mβ	A.m.Mγ	A.m.Mβ'^1^	A.m.Mβ'^2^	D.m. mβ	D.m.Mγ
D.m.Mγ	72/65	72/66	71/61	71/62		
D.m.Mβ	77/67	69/63	73/62	76/66		
A.g.Mβ	83/76	70/66	67/57	66/57	80/70	76/67
A.m.Mγ	73/67		69/57	68/54		
A.m.Mβ			69/60	64/56		

A.m.Mβ'^1^: own prediction

A.m.Mβ'^2^: database prediction

Penalties: bestfit: Gap weight: 1, length weight: 1, max. penalized length: 30

### The *Achaete-Scute *complex

Genes related to *achaete *or *scute *have been identified in a large number of species, from hydra [47] to mouse [30], and so we expect these also in the different insects. The *AS-C *was most intensely studied in various species of *Schizophora *flies, apart from *Drosophila *[28,37,48-53]. The number of genes varies between one and four, however, is not strictly correlated with the position in the phylogenetic tree. For example, *AS-C *of *Calliphora vicina *contains three genes, whereas other dipteran flies like *Drosophila *contain four. Two genes are found in the branchiopod crustacean *Triops longicaudatu*s [54] and only one in hydra [47]. In Dipteran flies the expression patterns of the proneural genes are largely varied. This is regulated by positional information through the *Iroquois Complex *and *pannier *and in addition by a transcriptional feed-back loop involving *AS-C *proteins. Eventually, neural precursors are selected by the repressive activity of E(spl) bHLH proteins [55,56]. Thereby, location and number of the large bristles on the notum is precisely controlled. The mosquito is covered with rows of large sensory bristle, where number and position varies between individuals [57]. This is in accordance with the fact that there is only one *scute*-like gene, *A.g.ash *that is expressed all over the presumptive notum in a modular pattern [37]. Recently it was shown that the *Anopheles A.g.ash *gene can mimic the endogenous *Drosophila *genes and that overexpression leads to many ectopic bristles [37].

Albeit the bristle pattern on the notum of different Drosophilids varies slightly, bristle number and position is highly stereotyped [58]. Therefore, it is not surprising to find the *AS-C *highly conserved within Drosophilids. Yet, the rate of change came unexpected and is quite remarkable outside of the bHLH domain. Compared to E(spl) bHLH proteins, those encoded by *AS-C *have a rather low degree of similarity, most notably Ac. In fact, the big flesh fly *Calliphora vicina*, which like *Drosophila *belongs to the *Schizophora*, is totally lacking the *ac *gene and is covered with bristles [51]. In agreement, we were unable to find *ac *in *Anopheles *or *Apis*, arguing for rapid evolution. The best conservation rate is found in Sc and L'sc suggesting high evolutionary pressure and maybe common ancestry. Not only the bHLH domain, but also two small stretches outside (aa 203; SPTPS in *D. melanogaster *L'sc) and also the C-terminus are of high similarity, the latter found identical in *Calliphora *[51]. Presumably these protein domains are of functional importance. Indeed, the C-terminus acts as transcriptional activation domain and is also used to recruit E(spl) bHLH proteins [36]. Although the alignments of the respective genes of honeybee and mosquito to *sc *and *l'sc *are very similar, the tendency goes to a closer relationship to *l'sc*. However, we propose that this gene pair arose by duplication in the course of Drosophilid evolution, such that we may be looking at a common ancestor in the other two species.

The rate of conservation is very limited for the Ase homologs. Decent conservation is found within the bHLH domain, and moreover, a further well-conserved box is present (NGxQYxRIPGTNTxQxL; x are differences between *A. gambiae *and *D. melanogaster*). This sequence is likewise detected in the Ase protein of *C. vicina*, which however shares many more similarities with D.m.Ase [51]. In *Apis*, there is no such conservation outside of the bHLH domain, which itself is highly diverged. The overall degree of conservation is so poor that further statements about the relationship are difficult. We argue that this gene represents *A.m.ase *by its close proximity to *A.m.ash*, although other interpretations are similarly possible. An analysis of its expression pattern in honeybee may help to solve these questions.

## Conclusion

We aimed towards an understanding of the evolution of *E(spl)-C *and *AS-C *complexes which in *D. melanogaster *comprise genes of apparent redundant functions. Our analysis covered insect species that belong to the orders Hymenoptera (honeybee) and Diptera and there to the suborders *Nematocera *(mosquito) and *Brachycera *(three species of the genus *Drosophila*) and thus spans an about 300 Myr window of evolution. We find that both *E(spl)-C *and *AS-C *expanded rather recently as they are only present in their nowadays complex structures in Drosophilids. In *Apis *and in *Anopheles*, we find very similar arrangements indicative of an ancient ur-complex. The *E(spl)-C *seems to have evolved from two genes, one HES-like and one Brd-like gene that are arranged in a head to head orientation. Both types of genes are responsive to Notch signalling in *Drosophila*. Our data suggest that the most ancient genes are *E(spl) *bHLH *mβ *and *E(spl) m*α from which the other *E(spl)-C *genes derived by duplication and subsequent change. Moreover, an *E(spl) *ur-complex is likewise detected in *Tribolium castaneum *that belongs to the order Coleoptera. In *Drosophila *the complex also gained unrelated genes like *m1 *and *gro*. The latter is highly conserved, however, located at different genomic positions. Whereas in *Anopheles *the ur-complex seems to exist in its original form, two additional *mβ*-like bHLH genes are found in the *Apis *genome that possess introns. These introns are at similar positions as the introns of two other HES-like genes, *dpn *and *h *which themselves are highly conserved in the three insect species, arguing for a common evolutionary history. Presumably, the introns are evolutionarily ancient as they are also found in the *C. elegans E(spl)/h *like gene *lin-22*. The *AS-C *seems to originate from a single *sc/l'sc *like bHLH gene and a second largely diverged bHLH gene that shares similarity with *Drosophila ase*. The high degree of variation in the latter makes it difficult to conclusively decide on the original arrangement of this gene complex.

## Methods

### Databases

*Drosophila melanogaster *gene and protein sequences were accessed in FlyBase [31]. The *D. pseudoobscura *database is found at the Human Genome Sequencing Center [59]. The genome of *D. virilis *is sequenced by Agencourt Bioscience Corporation and can be downloaded [60] or searched [61]. The *Apis mellifera *and *Anopheles gambiae *databases can be accessed with the Ensembl genome browser [62]. The honeybee genomic sequence is also available at the Human Genome Sequencing Center [59] and the EST sequences were obtained from the honeybee brain EST project [63]. Table 2 lists accession numbers of genes, contig numbers and positions of genes therein as well as accession numbers to gene predictions. Due to updates of the databases, gene annotation and names may be different now than reported here.

### Sequence analysis

Sequences were downloaded and further studied applying the HUSAR programs of the Deutsche Krebsforschungszentrum [64,65]. Genomic DNA was translated with MAP. BESTFIT and GAP programs were used for alignments and calculation of similarity and identity scores. DOMAIN SWEEP was applied to define bHLH and orange domains, respectively. The amphipathic α-helices were predicted and drawn using WHEEL.

### *D. pseudoobscura *sequence acquisition

The annotation of the *D. pseudoobscura *database is well advanced. TBlastN searches starting from FlyBase [31] or using *D. melanogaster *genes allow easy access to both *E(spl)-C *and *AS-C *gene complexes. The database also gives information on transcript length, structure and orientation.

### *D. virilis *sequence acquisition

Searches were done with BlastN. However, the *D. virilis *database allows this scan only with DNA against DNA. We therefore searched first for a characteristic part of a *D. melanogaster *gene (i.e. the bHLH domain) using lowest percent identity option (75% identity) to find the respective *virilis *ortholog. We then used the FIND program to identify the respective contig containing these sequences within the downloaded genomic sequence. Subsequently, the entire contig was translated in all six possible reading frames and manually analysed for *E(spl)-C *and *AS-C *genes and proteins. The complete *E(spl)-C *was located in one contig. To identify the whole sequence of the *AS-C*, three overlapping contigs had to be investigated. However, the contigs covering the *AS-C *still contained many large unsequenced or uncertain stretches. Therefore, the exact size of the complex could not be defined. The sequence gaps do not affect the coding sequences of the studied genes.

### *A. gambiae *and *A. mellifera *sequence acquisition

The other databases were screened with *D. melanogaster *protein sequences for members of *E(spl)-C *and *AS-C *against genomic DNA. After detecting convincing similarities, the surrounding genomic DNA was downloaded for further studies with the HUSAR programs. Most of the genes that we describe here have also been annotated as transcribed regions. However, the majority of the database predictions were either incomplete or inconsistent. For our predictions, we carefully analysed genomic DNA and possible coding sequences. All six reading frames were searched for bHLH domain sequences and in the case of the *E(spl)-C *bHLH proteins also for WRPW motifs. Predicted introns were scanned for open reading frames and their borders reinvestigated. Exon/intron boundaries were defined by obeying to the GT AG rule. Afterwards it was analysed whether the newly predicted introns affected the open reading frame.

### Classification of gene homology

Gene homology and orthology was classified based on sequence identity with the respective *D. melanogaster *protein. In the studied *Drosophila *species all analysed genes have been identified in a 1:1 ratio and could therefore be classified as orthologs. The degree of similarity and identity between two related proteins was determined with the BestFit program [64]. Comparison of proteins from the three *Drosophila *species was done under the pre-configured standard conditions of the program (Gap weight: 8, length weight: 2). A TBlastN search in the *Apis *and *Anopheles *databases always returned several sequences, and we analysed up to 15 of the best hits. If the hit was in a region without predicted gene, we translated the respective sequence and analysed the open reading frames manually. They were analysed for expected protein motifs, like bHLH, orange or WRPW domain. The predicted protein sequences were then used for a BlastP search in FlyBase. In case of a 1:1 relationship, the genes were classified as orthologs. Since there are several *E(spl)-C *bHLH and *AS-C *bHLH genes in *Drosophila*, a 1:1 allocation was not possible, therefore, we classified them as homolog (see results). Protein sequences derived from the *Apis *or *Anopheles *genome projects are so diverged, that standard conditions only align the best conserved domains. We therefore changed the conditions to Gap weight: 1, length weight: 1, max. penalized length: 30. These relaxed conditions had little influence on either alignment of well-conserved sequences, the similarity or identity values. For example, the D.m.Mβ and D.v.Mβ orthologs share 89% identity under stringent conditions versus 93% identity under relaxed conditions. The reduced stringency however, allowed an alignment of the entire protein sequence also of the diverged proteins with the consequence that the identity values increased considerably compared to standard conditions. For example an alignment of A.g.Ash (371 residues) with D.m.L'sc (257 residues) under standard conditions aligns only the residues A.g.Ash 50–233 with D.m.L'sc 40–218 with an identity of 46%. Under relaxed conditions the whole protein sequences align with 64% identity.

## Authors' contributions

RS has contributed substantially to data acquisition and participated in sequence alignments. DM designed the study, acquired and analysed the data and drafted the manuscript. Both authors have read and approved the final manuscript.
